# Low-Cost Wearable Fluidic Sweat Collection Patch for Continuous Analyte Monitoring and Offline Analysis

**DOI:** 10.1021/acs.analchem.2c01052

**Published:** 2022-04-29

**Authors:** Annemarijn S. M. Steijlen, Kaspar M. B. Jansen, Jeroen Bastemeijer, Paddy J. French, Andre Bossche

**Affiliations:** † Faculty of Electrical Engineering, Mathematics & Computer Science, Delft University of Technology, Mekelweg 4, Delft 2628 CD, The Netherlands; ‡ Faculty of Industrial Design Engineering, Delft University of Technology, Landbergstraat 15, Delft 2628 CE, The Netherlands

## Abstract

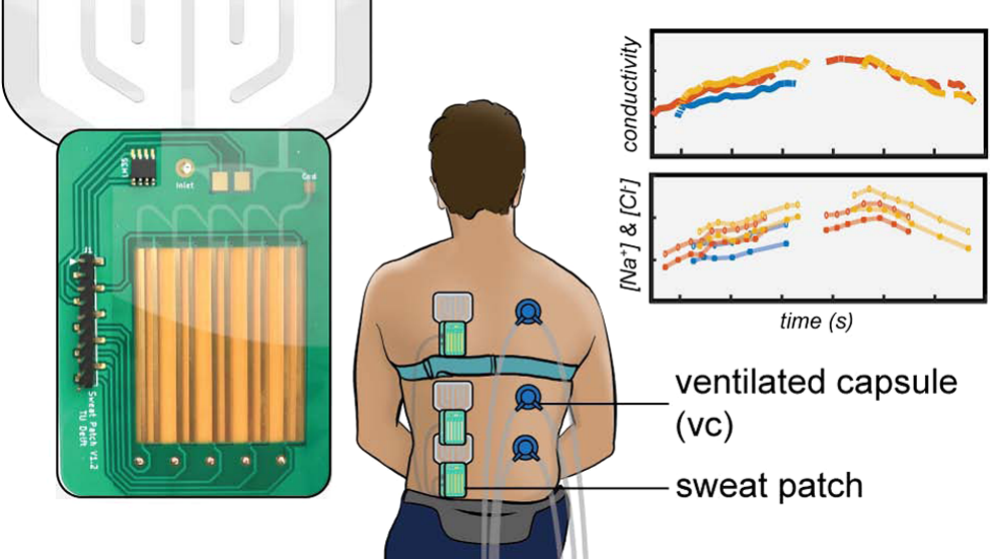

Sweat sensors allow for new unobtrusive ways to continuously monitor an athlete’s performance and health status. Significant advances have been made in the optimization of sensitivity, selectivity, and durability of electrochemical sweat sensors. However, comparing the *in situ* performance of these sensors in detail remains challenging because standardized sweat measurement methods to validate sweat sensors in a physiological setting do not yet exist. Current collection methods, such as the absorbent patch technique, are prone to contamination and are labor-intensive, which limits the number of samples that can be collected over time for offline reference measurements. We present an easy-to-fabricate sweat collection system that allows for continuous electrochemical monitoring, as well as chronological sampling of sweat for offline analysis. The patch consists of an analysis chamber hosting a conductivity sensor and a sequence of 5 to 10 reservoirs that contain level indicators that monitor the filling speed. After testing the performance of the patch in the laboratory, elaborate physiological validation experiments (3 patch locations, 6 participants) were executed. The continuous sweat conductivity measurements were compared with laboratory [Na^+^] and [Cl^–^] measurements of the samples, and a strong linear relationship (*R*^2^ = 0.97) was found. Furthermore, sweat rate derived from ventilated capsule measurement at the three locations was compared with patch filling speed and continuous conductivity readings. As expected from the literature, sweat conductivity was linearly related to sweat rate as well. In short, a successfully validated sweat collection patch is presented that enables sensor developers to systematically validate novel sweat sensors in a physiological setting.

Continuous health monitoring can contribute to the prevention of chronic diseases by creating awareness about lifestyle and by stimulating physical activity.^1,2^ Furthermore, it may support in the prevention of injuries^3,4^ and it can contribute to the effective and efficient treatment of diseases.^5,6^ Common health monitoring devices include heart rate monitors and movement sensors. Recent advances in wearable sweat sensor systems show great potential to add new physical and chemical information about a person’s health status. Sweat samples can be collected unobtrusively and continuously, which are the main advantages compared to the widely used blood tests. The unobtrusive nature of sweat sensing and its potential use in real-time health monitoring motivated researchers to develop electrochemical sweat sensors to monitor sweat constituents. Literature reviews show a vast number of recently developed sweat sensors.^7−11^

Although sweat provides ways to unobtrusively monitor biomarkers, measuring sweat constituents introduces new challenges that are not present in blood sampling. First, sweat rates vary over time and influence the composition of sweat. Second, sweat rate and composition differ per body location. Third, sweat rates can be very low, which results in low sample volumes too. Fourth, skin and sweat gland metabolism and skin contaminants influence the composition of sweat. And lastly, sample collection is difficult due to quick evaporation and the irregular skin surface.^12^ Furthermore, scientific knowledge about the physiological mechanisms of sweating and how sweat analyte levels relate to blood levels is limited.^13^ During strenuous exercise and/or exposure to hot environments, body core temperature rises. Evaporation of sweat from the skin plays a critical role in regulating body temperature.^14^ The thermoregulatory function of sweating is well-accepted, but the physiological knowledge about using sweat constituents, *i.e.*, electrolytes and metabolites, as a biomarker is limited. For most constituents in sweat, correlations between concentrations in blood and concentrations in sweat are not known. For sodium and chloride, with concentrations in sweat ranging from 10 to 100 mM, secretion mechanisms are known.^15^ Na^+^ and Cl^–^ ions are secreted in the secretory coil of the sweat glands and partially reabsorbed in the duct. The number of reabsorbed ions has a direct relation to the sweat rate. When sweat rates increase, a lower percentage of ions is reabsorbed and the absolute concentration of these ions in sweat increases.^14,16^ Several articles suggest that an increase in Na^+^ or Cl^–^ levels can be used as a biomarker for dehydration.^17,18^ There are also researchers who state that [Na^+^] and [Cl^–^] loss can indicate electrolyte imbalance.^19^ However, in physiological literature, even for these most available ions in sweat, equivocal and even contradictory results can be found.^20^ The main reason for these existing gaps in literature is the lack of standardized methods for chrono-sampling of sweat.^21^ Sweat samples are mostly collected with simple devices, such as absorbent patches and the Macroduct sweat collector.^22^ Chrono-sampling with these devices requires a lot of repetitive work. This results in a limited number of samples.

To facilitate chronological sampling of sweat and to enable continuous sweat monitoring, many new sweat sensor systems have been developed recently. Most sensors focus on continuous measurement of a sweat constituent, such as electrolytes and metabolites, with a miniaturized wearable device. Some researchers focus on the microfluidic system that enables capture and transport of the sweat to the sensor,^23−25^ while other researchers focus on the fabrication and improving the specifications of the sensor itself.^26,27^ In particular, a lot of research is being executed in optimizing the sensitivity, selectivity, and durability of the electrode materials.^28−30^ Additionally, significant progress has been achieved in sweat rate measurement^31,32^ and design integration of the fluidic system and the sensors.^25,33,34^ These novel systems may support physiologists in their research to find sweat biomarkers, but there are two major limitations. First, the fabrication of the systems has high complexity and requires advanced equipment and expensive materials. Second, the new systems are validated in an exercise setting with a very limited number of participants. Interpretation of the data and comparing them against results from other articles is a challenge, because protocols are not standardized, and in most cases, no sweat reference measurements are performed during the exercise.

The aim of this study is to develop and present a sweat collection patch that enables continuous *in situ* monitoring of sweat composition and controlled sampling of sweat in reservoirs for offline analysis. The patch distinguishes itself from other recently developed patches by its easy and low-cost fabrication. Additionally, the sweat collection area and sample volumes are generally larger (typically >10 times) than in previously presented chronological sweat collection systems,^35,36^ to increase the number of samples in time and minimize the effects of skin contamination and increase the reliability of the measurements. Patches that collect relatively large samples do exist, but these patches comprise a single reservoir.^37,38^ The new patch consists of an analysis chamber that hosts a conductivity sensor for measuring ionic content continuously. Once the sweat passes this chamber, it flows into a sequence of reservoirs, which include level indicator electrodes. The level indicator electrodes measure the filling speed, which is dependent on the sweat rate. The sweat in the reservoirs can be analyzed offline. In this way, *in situ* sweat composition measurements of the patch can be directly compared with lab measurements. The new sweat patch is made with accessible fabrication techniques. This means that the patch can be easily reproduced, and other novel electrochemical can be placed in the analysis chamber to perform controlled validation experiments in a physiological setting.

To test the performance of the collector patch, lab validation experiments are executed, followed by physiological experiments in a climate chamber. In these experiments, the relationship between *in situ* sweat conductivity measurements and [Na^+^] and [Cl^–^] of the samples, measured by ion chromatography in the lab, is researched. Thereafter, sweat rates measured with a ventilated capsule measurement system are compared with the conductivity measurements and filling rate measurements from the patch.

## Materials and Methods

### Sweat Patch

**Figure 1:**
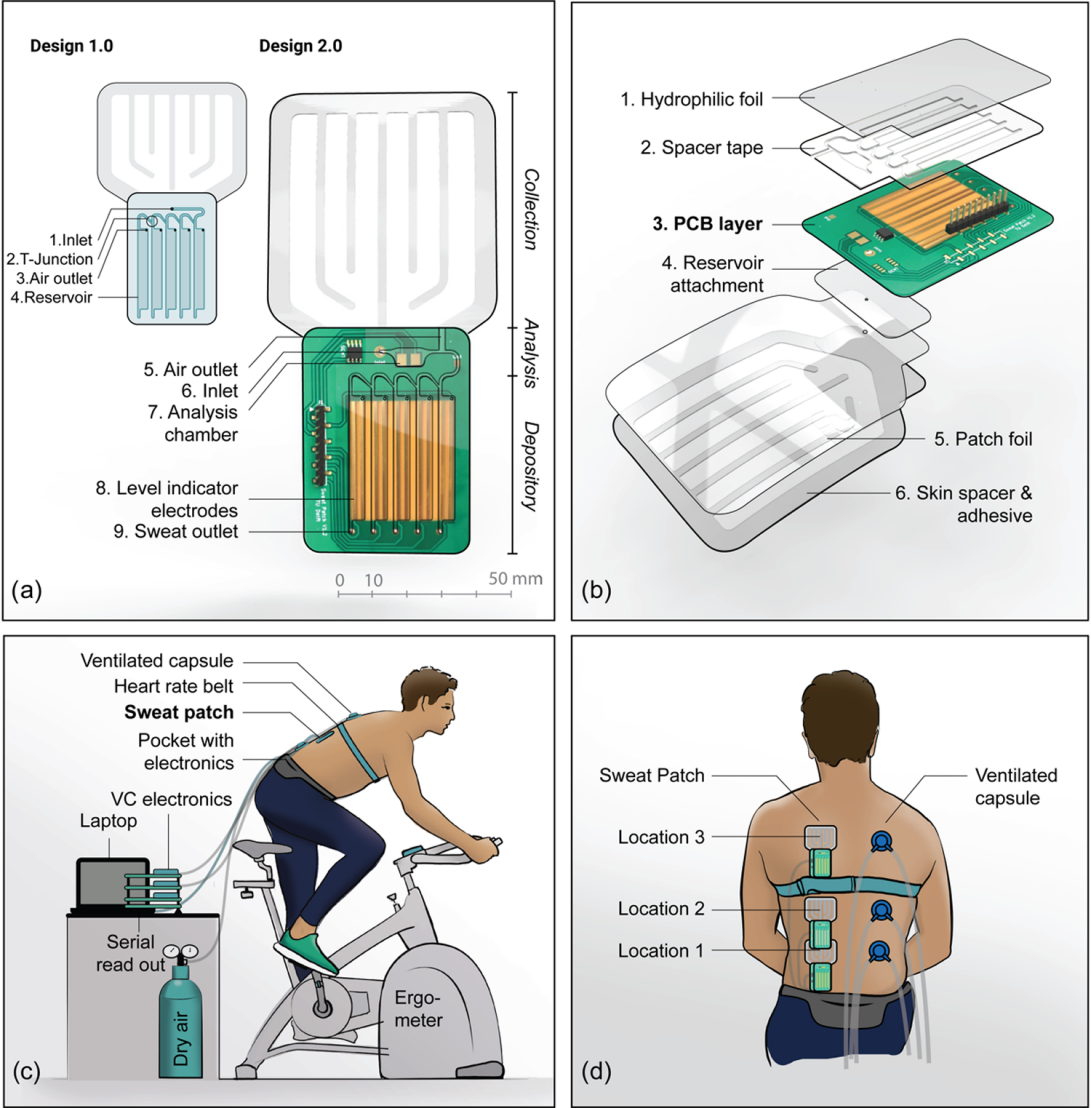
(a) Front view of the design of the preliminary collector patch (left) and the new patch with integrated sensors (right). An extra reservoir is added near the inlet (No. 7). All sweat will pass this reservoir. Electrodes in this reservoir measure sweat conductivity. Level indicator electrodes are placed in the bottom reservoir sequence. (b) Exploded view of the new patch. (c) Setup for physiological tests with the new sweat patch. The patch is placed at the subject’s back and electronics are placed in a waist pocket. For the ventilated capsule system, dry air flows through a capsule at the subject’s back and the humidity and temperature are measured by the sensors that are connected to the outlet of the capsule. (d) Measurement locations of the patches (left) and the capsules (right).

To test the flow characteristics, a preliminary collection patch was developed and tested without integrated electronics. As can be seen in Figure 1a, the patch consists of a funnel-shaped structure that guides the sweat to the collector inlet. By capillary and gravitational forces, the sweat will flow to the first reservoir. Once this reservoir is filled, the new fluid will pass to the subsequent reservoir. The collector is fabricated from two layers of hydrophilic film (Visgard 275, a poly(ethylene terephthalate) (PET) film with a polyurethane (PU) coating^39^) and a double-sided adhesive (3M 1522, a PE tape with an acrylate adhesive^40^) in between. The structure of the channels and reservoirs in the acrylate adhesive and the contours of the hydrophilic foil are cut with a CO_2_ laser system (Merlin Lasers, Lion Laser Systems, The Netherlands). Simulations and experiments with a syringe pump proved that the reservoirs fill consecutively, and that sweat inflow is not significantly inhibited by the resistance of the channels. This first version of the patch was presented in a previous article in more detail.^41^ The patch is designed to be placed at the back of a person. This version has a collection surface of 40 cm^2^ and can collect 5 samples of 130 μL. Sample volume and collection surface can be adjusted to the type of physiological experiment.

After testing the first patch in a physiological setting, a redesign was made (Figure 1a). An extra chamber, the analysis chamber (Figure 1a, No. 7), was placed near the inlet of the reservoir system. This chamber has rounded corners so that there is a constant renewal of sweat. Preferred marker-specific electrodes can be placed in this reservoir. The extra air outlet in the top (Figure 1a, No. 5) ensures that electrodes stay covered with sweat. In this design, the back foil layer of the reservoir system was replaced with a thin printed circuit board layer (0.6 mm) with gold electrodes. The gold electrodes are located in the new chamber to measure the sweat conductivity over time. Furthermore, electrodes were added in the sequence of reservoirs to measure the filling rate which depends on the sweat rate (Figure 1a, No. 8). Two parallel elongated gold electrodes were positioned in each of the five reservoirs, which allows us to measure a conductance change when the reservoirs are filling up. A temperature sensor (LM35, Texas Instruments) was placed at the PCB as well. Since the PCB materials are less hydrophilic, contact angle measurements and new syringe pump experiments were performed to test whether the new collector fills at the same rate as the sweat rate. Contact angles of the gold electrodes and the solder mask are 69.8 and 73.1°, respectively, while the contact angles for the adhesive tape and the hydrophilic film are 92.2 and 50.5° (measured with water at *t* = 10 s after contact with the surface, with optical tensiometry, KSV Instruments Ltd.).

For both sensors, the AD5933 impedance converter (Analog Devices) is used to measure the impedance. The integrated circuit contains a frequency generator (up to 100 kHz, Vpp: 0.2–2 V), and a 12-bit analog-to-digital converter (ADC) to sample the impedance. The chip also holds a digital signal processor (DSP) that performs a discrete Fourier transform to return the magnitude of the impedance and the phase of the impedance at the defined output frequency. An 8-channel multiplexer (ADG1408, Analog Devices) is used to switch between the conductivity sensor, sweat rate sensors, and a calibration resistor. The excitation voltage is set at 0.2 Vpp to prevent saturation of the ADC at lower resistances, and the frequency is set at 80 kHz to minimize double-layer effects. The AD5933 and the multiplexer are placed in a pocket that can be carried around the waist. The patch is connected to the readout electronics via simple header pins. Figure 1b shows an exploded view of the patch design.

To calibrate the conductivity sensor in the lab, different solutions of NaCl (from 10 to 150 mM) were placed in the top reservoir of a patch. The impedance was measured for each solution to find the relationship between the NaCl concentration and the conductance in the analysis chamber. The functioning of the level indicator electrodes was tested using the syringe pump (KD Scientific 200). Pump rates ranging between 12 and 60 μL/min were chosen. For patches with a collection surface of 40 cm^2^, this translates to sweat rates of 0.15 to 1.5 mg/(cm^2^ min). For these experiments, a NaCl solution of 75 mM was used.

### Reference Measurement

To research the performance of the sweat collector patch in the physiological setting, two types of reference measurements were performed. First, the continuous *in situ* conductivity measurements were compared to ion chromatography measurements of the samples that were collected and stored in the sequence of reservoirs. Second, to investigate whether sweat conductivity measurements and patch filling rate relate to the actual sweat rate, these measurements were compared against sweat rate measurements with the ventilated capsule technique.

### Ion Chromatography

This paragraph describes the procedure for offline chemical analysis. In each collection patch, tiny outlets are made in the PCB layer at the bottom of each reservoir (Figure 1a, No. 9). These holes are covered with adhesive tape during collection. Once the patch is removed, the tape can be removed and the sweat can be pushed into a vial by injecting air in the reservoir with a syringe at the air inlet. The sweat volume was weighed and diluted with ±3 mL of ultrapure water, after which the vials are stored at −20° C and analyzed in the laboratory. In this study, we focused on the analysis of electrolytes using ion chromatography/high pressure liquid chromatography (IC/HPLC). [Na^+^], [Cl^–^], and [K^+^] were measured. For the anions, a Metrohm 881 Anion system with a Metrosep A supp 5–150/4.0 column was used. The cations were analyzed with the 883 Basic IC plus system and a Metrosep C6–150/4.0 column (Metrohm, Switzerland). A more detailed description of this method can be found in Steijlen et al.^41^

### Ventilated Capsule Measurement

The most common methods for measuring local sweat rate in a lab setting are the ventilated capsule (VC) technique and the absorbent patch technique.^42^ Using absorbent patches requires a lot of repetitive work, and a limited number of samples can be collected over time. Therefore, the VC measurement is more suitable to measure sweat rate continuously.^43^ Capsules with a collection surface of 5.3 cm^2^, made from a flexible three-dimensional (3D)-printed photopolymer on a Connex 3 3D printer (Objet350, Stratasys Ltd., Israel), were placed on the skin. The capsule is connected to a dry air cylinder, and air flows through the capsule at a volumetric flow rate between 0.1 and 1.2 L/min. The flow rate is measured with a variable area flow meter (Key Instruments). A humidity sensor and temperature sensor (HDC1080, Texas Instruments) are connected to the outlet of the capsule. The sensors are controlled with an MSP430 microcontroller (Texas Instruments). We used the Antoine equation in the conversion of relative humidity to absolute humidity. Knowing the dry air flow rate, absolute humidity, and sweat collection surface, the sweat rate was calculated.

Lab measurements were executed to test the performance of our VC system. A detailed description of these experiments and the results can be found in the Supporting Information (S1). To test whether the VC system measures all sweat in the capsule and to test if there are no leakages, a predetermined amount of water was placed in a closed capsule and the total amount of sensed water was calculated by numerical integration of the evaporation rate measurements. It was concluded that the deviation was adequately small (1–5%). Furthermore, the response and recovery time of the VC system were calculated. At a dry air flow rate of 1.2 L/min, the response time is 118 s and the recovery time is 353 s. Because sweat rates will always increase or decrease gradually, the actual response time will be even faster in a physiological setting. This means that the VC system can be used for continuous monitoring of sweat rate. The maximal sweat rate that can be measured with the current system is 2.75 mg/cm^2^/min.

### Physiological Experiments

Healthy recreational athletes (3 female and 3 male participants 20–30 years) cycled (Lode Excalibur, The Netherlands) in a climate chamber (b-Cat, The Netherlands) that was set to 33 °C and 65% relative humidity. Figure 1c shows the setup of the physiological experiments. The patches were placed at three locations at the back on the left side of the spine. Three ventilated capsules are attached to the right side of the spine at the same heights, as can be seen in Figure 1d. The locations were chosen based on the study of Smith and Havenith, which showed relatively high sweat rates at the back and similar sweat rates at the left and right side of the sagittal plane.^44^ The capsules are placed from the start of the exercise and the dry air flow rate was set at 1.2 L/min to allow for the expected high sweat rates in this climate. Heart rate was measured with a heart rate belt (Polar H10, Finland), and body core temperature was measured with a rectal temperature probe (Yellow Springs Instruments) during the experiments. The participants cycled 30 min at 40–50% of their maximum heart rate (HRmax), followed by 20 min at 60% HRmax and 20 min at 70% HRmax. Afterward, the participants stopped cycling and a cooling down period of 20 min at the stationary bike was included. Sweat tests were approved by the Human Ethics Research Committee of Delft University of Technology. The initial 30 min included the 20 min wash out period of the skin.^20^ The first sweat patch was placed after this initial 30 min. Once all reservoirs were filled with sweat, the patch was replaced with a new one. Collectors with 10 reservoirs of 70 μL and a collection surface of 20 cm^2^ were used.

## Results and Discussion

### Characterization of the Collection Patch

**Figure 2:**
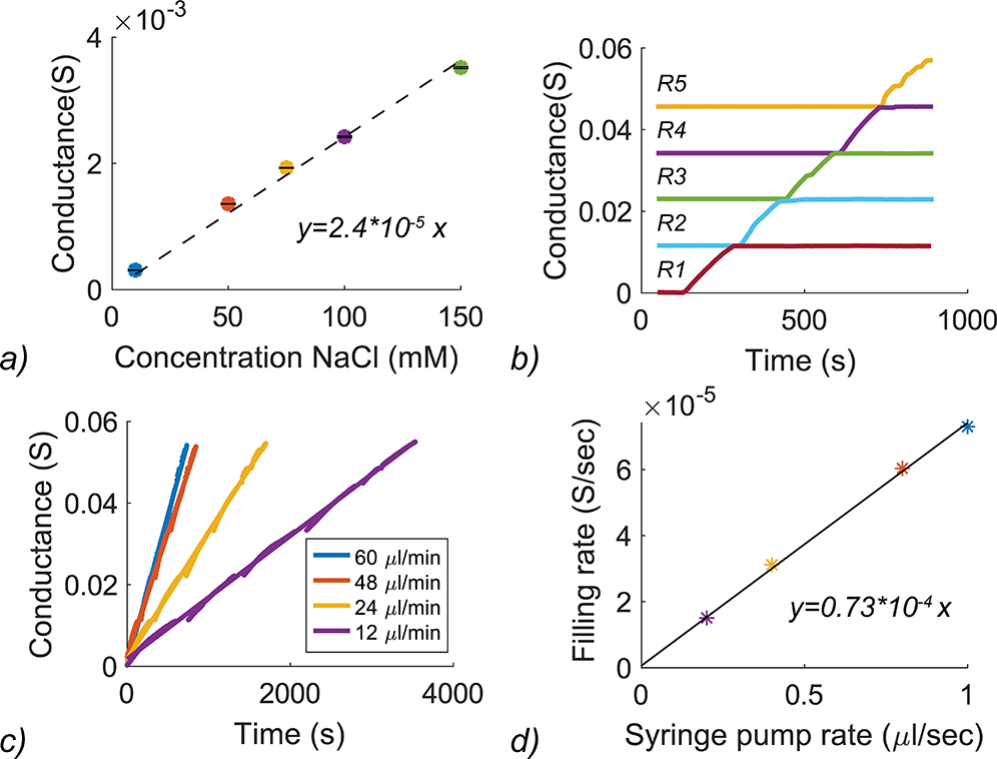
(a) Conductance measurements in the analysis chamber that is filled with different solutions of NaCl. (b) Results of a filling rate measurement when the syringe pump is set at 12 μL/min. Each color represents the measurement of a separate pair of electrodes in a single reservoir. Cumulative results show the course of the filling process. (c) Conductance over time at 4 different pump rates. (d) Relationship between the syringe pump rate in μL/s and the filling rate in S/s.

During the patch characterization experiments, two topics were addressed. First, it was researched if the conductivity sensor in the analysis chamber can measure NaCl concentrations in the desired range. Solutions of NaCl (10–150 mM) were injected in the analysis chamber. By performing a frequency sweep (from 5 to 100 kHz), when measuring the different solutions, it was found that at 80 kHz, there were no significant influences of stray capacitances on the measurements. Figure 2a shows the relationship between the conductance measured with the conductivity sensor in the top reservoir and the concentration NaCl of the standard solutions. A linear relationship was found, and the sensitivity of the sensor system was 24 μS/mM. Using the molar conductance of NaCl in water,^45^ a cell constant of 5.223/cm was calculated.

Second, the performance of the collection system was tested with the level indicator electrodes. It was researched whether the reservoirs fill consecutively, and if the conductance change measured with the level indicator electrodes are linearly related to the volumetric flow rate settings of the syringe pump. To test the consecutive filling of the reservoirs, the syringe pump was set at a flow rate of 12 μL/min. Figure 2b shows the results of the measurement of the separate electrode pairs in pre-wetted reservoirs. Once a previous reservoir is filled, the total conductance is added to the next measurement to see the course of the filling process. Figure 2c shows that the collectors fill at a constant speed at increased pump rates as well and a linear relationship between the different pump rates and the filling rate in Siemens per second was obtained (Figure 2d).

### Physiological Experiments

**Figure 3:**
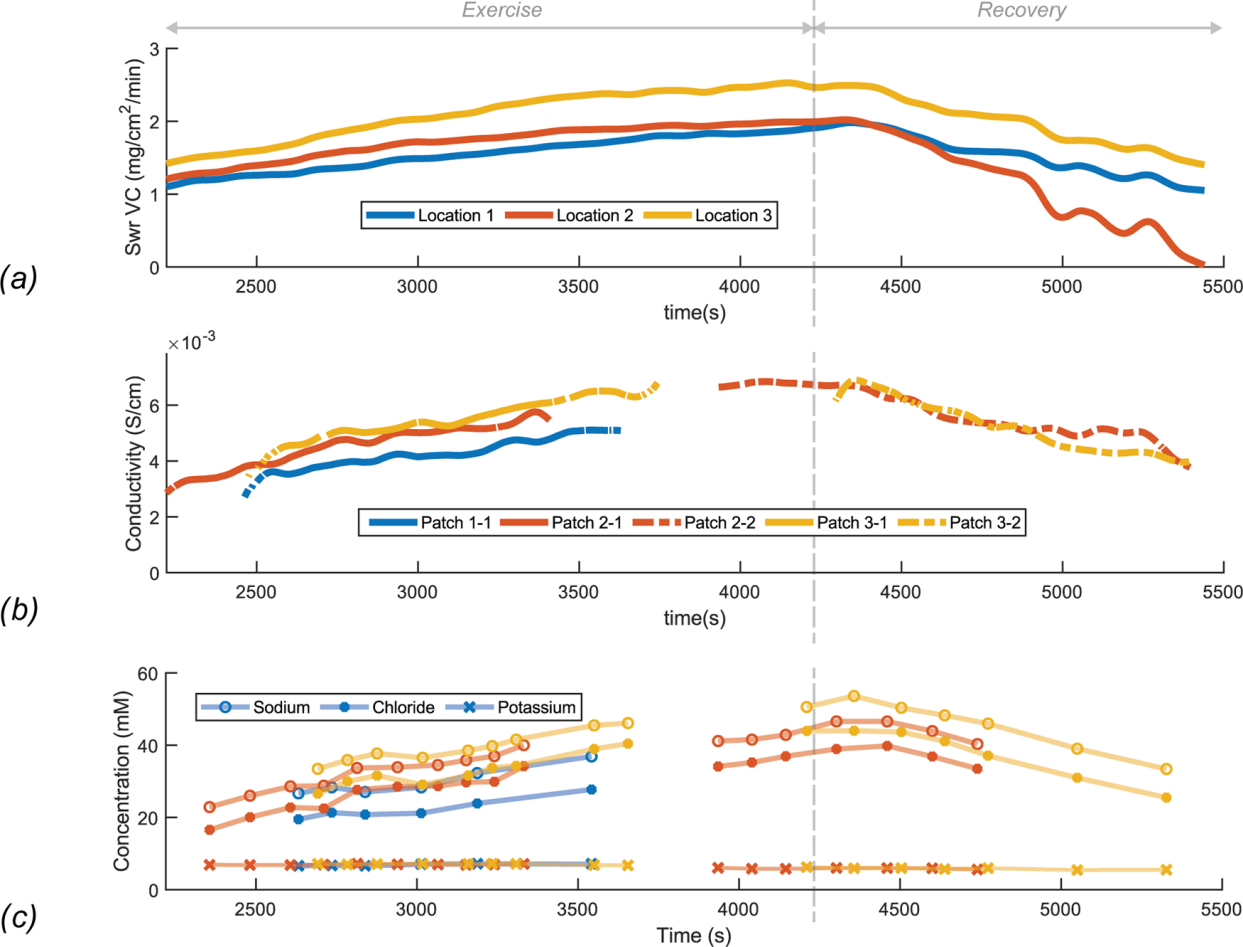
(a) Continuous sweat rate measurement from the VCs placed at the 3 locations at the back of participant 1. The incremental exercise was performed until *t* = 4200 s, followed by a recovery period of 1200 s. The patches were placed at *t* = 1800 s. (b) Conductivity measured in the analysis chamber of the patches at the 3 locations of participant 1. Gaps are due to patch replacement (*e.g.*, patch 2-1 is replaced by patch 2-2). (c) [Na^+^], [Cl^–^], and [K^+^] data from chromatographic analysis of the samples from the individual reservoirs. Timestamps were derived from the filling rate measurements. Lines indicate that the samples are from the same patch.

All six participants completed the protocol in the climate chamber. For most participants, the patches started filling typically around 8 min after placement. Once a patch was completely filled, it was immediately replaced by a new one. Sweat rates highly varied among participants. For one participant at 2 locations at the back, three patches of 10 reservoirs were completely filled, while for another participant, only 3 reservoirs were filled during the entire exercise. Body core temperature increased 1.03 ± 0.72° during the exercise. Figure S2 shows real-time rectal temperature and heart rate of participant 1 during the exercise. A picture of the experimental setting is presented in Figure S3. Figure 3a shows the sweat rate over time measured with the ventilated capsule system for the three different locations at the back of participant 1. The sweat rate was calculated using the air flow rate, humidity, and temperature measurements. Data were filtered with a Savitzky–Golay sliding window filter^46^ using a second-order polynomial and a window size of 150 data points. The sample frequency of the measurements was 1 Hz. In Figure 3b, the conductivity measurements in the patch analysis chamber at the same locations on the left side of the back of participant 1 are plotted. Low conductance values (<0.5 × 10^–3^ S) and abrupt changes in conductance, attributed to air bubbles in the system, were removed. Subsequently, a similar sliding window filter was used to remove high-frequency noise components (window size: 50, sample rate: 0.3 Hz, second-order polynomial). Unfiltered data can be found in the appendix (Figure S4). Temperature changes over time varied from 0 to 1.5 °C for each patch from the start of the filling process. The temperature coefficient of conductivity of electrolyte solutions is around 2%/°C at 25 °C.^47^ Thus, the conductivity change due to temperature effects would be estimated between 0 and 3% maximum. Given that the conductivity increased more than 100% during the course of the exercise of participant 1, temperature changes have limited influence.

It can be noted that all 3 graphs from participant 1 show a similar trend during the exercise. The sweat rate increased as the heart rate increased. After a few minutes from the start of the cooling down period *t* = 4200 s, the sweat rate decreases again. When the sweat rate increases, a lower percentage of ions will be reabsorbed by the sweat duct.^14^ Therefore, the conductivity measurement shows an increasing trend when the sweat rate increases. This corresponds to the ion chromatography results from the samples that were collected in the reservoirs. In Figure 3c, it can be seen that [Na^+^] and [Cl^–^] increase when the participant exercises, and when the participant stops exercising, the concentrations decrease again. [Na^+^] are on average 6.84 ± 0.98 mM higher than [Cl^–^]. This difference is presumably being caused by the abundant presence of other negative ions in sweat such as lactate and bicarbonate. [K^+^] concentrations show a slightly decreasing trend during the entire exercise. For K^+^, secretion mechanisms are partly known and probably these ions are not reabsorbed in the duct and therefore not influenced by changes in sweat rate.^13^ The results from the other participants showed corresponding trends for the three different measurements methods similar to the results presented in Figure 3.

Parrilla et al. also compared the results of newly developed sweat sensors during physiological tests with ion chromatography measurements.^48^ However, because they used the labor-intensive absorbent patch method and they used a different physiological protocol, the number of samples that were collected over time were limited to 1 for every 10–12.5 min. Ohashi et al. recently presented a new sweat collection system with micropumps for chronological sweat sampling, which proved to collect a sample approximately every 5 min.^49^ Although a larger sequence of samples can be collected, samples are highly diluted (typically four times) and the sampling requires a strict protocol during the tests.

### Sweat Conductivity and [Na^+^] and [Cl^–^]

**Figure 4:**
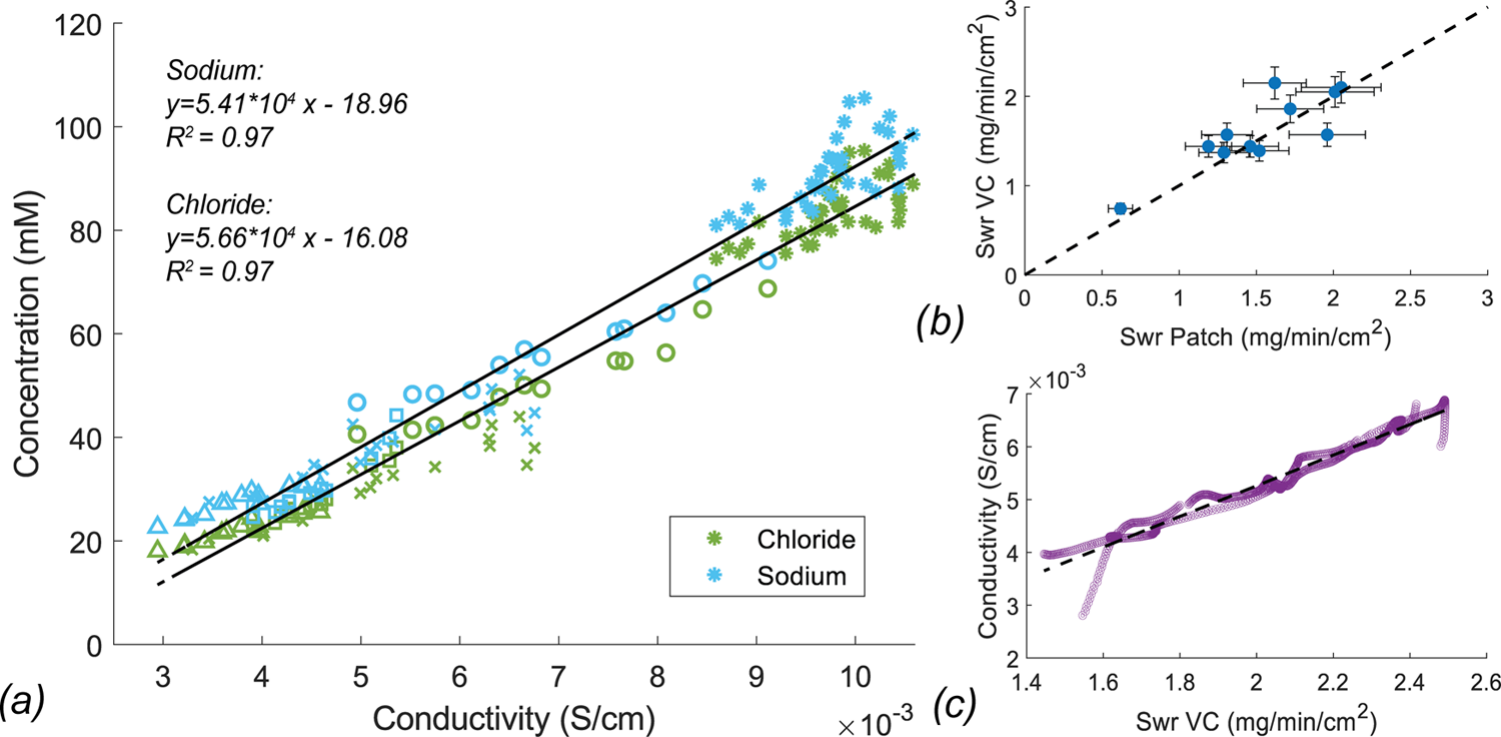
(a) [Na^+^] and [Cl^–^] plotted against conductivity of all samples (*n* = 97) of the physiological experiments. The different marker types represent the different participants. (b) Sweat rate of the VC plotted against the sweat rate of the patch. The dashed line represents the identity line. (c) Relationship between sweat conductivity and sweat rate of the VC at the upper back of participant 2.

In this study, it was aimed to test the performance of the sensor patch *in situ* by validating whether the lab measurements of the samples from the reservoirs can be used as a reference for the sensor in the analysis chamber. Therefore, a comparison between all ion chromatography results and conductivity measurements was made, by investigating the relationship between sweat conductivity and [Na^+^] and [Cl^–^] in sweat. First, the time intervals of the filling process of each reservoir were identified by detecting the starting times and end times of filling each reservoir. Subsequently, the time that it takes for the sweat to move from the top reservoir to the bottom reservoirs was subtracted from the time intervals. The mean conductance values of the top electrodes at the resulting time intervals were compared with the ion chromatography results. Because each electrode pair covers two reservoirs, concentration levels of two samples were averaged. In Figure S5, the results, including an error estimation of the three locations for each participant, can be found. A large range of concentrations was measured (from 18 to 105 mM). Participants that reached higher sweat rates also had significantly higher sweat Na^+^ and Cl^–^ concentrations. Figure 4a shows all measurements in one graph. [Na^+^] and [Cl^–^] are plotted against conductivity, and a strong linear relationship (*R*^2^ = 0.97) for both ions was found.

From these results, it can be concluded that the collector patch showed good performance because the continuous measurements in the analysis chamber were well related to the offline measurements of the two most abundant ions in sweat. Furthermore, it can be concluded that a 2-point AC conductivity measurement with gold electrodes can be used to obtain [Na^+^] and [Cl^–^] levels with good accuracy in these physiological conditions. Because of the higher stability and less complex fabrication process of the electrodes, conductivity sensors might be a more accessible choice than potentiometric sensors^50−52^ for integration in smart sweat patches that are aimed to monitor [Na^+^] and [Cl^–^] levels of healthy individuals. To find out if conductivity measurements can be used to obtain [Na^+^] and [Cl^–^] concentrations in other physical or specific dietary conditions, further research is required.

### Sweat Conductivity and Sweat Rate

The filling rate of the reservoirs of the patch can possibly be used as an indicator of the sweat rate. Therefore, it was researched whether this filling rate correlates well with the sweat rate derived from the VC technique. To obtain the actual filling speed from the level indicator readings, several data processing steps are required. The conductance will increase during the course of the filling process, but ionic content changes influence the conductance measurement as well. The cumulative average of the conductance data from the analysis chamber is used to compensate for the change in sweat conductivity due to ionic content change. eq 1 shows the relationship between the level indicator conductance (*C*_2_) and the cumulative average of the conductance in the analysis chamber (*C*_1_).
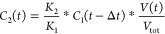
1in which *K*_1_ and *K*_2_ denote the cell constant in the analysis chamber and the cell constant of the level indicator electrodes, respectively. *V* is the current volume, *t* is time, and *V*_tot_ is the total volume of the sweat that is covered by 1 level indicator electrode pair. A time delay (Δ*t*), which is the time that it takes before incoming sweat at the inlet reaches the reservoirs, is included as well. In Figure S6, a graph of the filling rate (volume plotted against time) of two patches can be found. The abrupt changes in conductance over time are caused by droplet formation, and a linear fit was made to derive the average filling rate of a collector. The slope of this fit, or the filling rate, is plotted against the average VC sweat rate. For one participant, who had exceptionally high sweat rates, sweat rates were above the maximum evaporation rate of the system. So these data were excluded. Patches that showed leaks or patches that collected less than 4 samples were excluded as well. As can be seen in Figure 4b, the data of both techniques correspond reasonably well. However, a wider range of sweat rate measurements should be performed to draw a solid conclusion about the correlation. The main source of error appeared to be caused by the reading accuracy of the flow meters of the ventilated capsule system. Reading errors can result in an offset of maximum 8.3% of the measured sweat rate. These errors are represented by the horizontal error bars in Figure 4b. Furthermore, errors in the filling rate occur when small air bubbles are enclosed in a reservoir. The *R*^2^ value relative to the regression line of the best fit is 0.71 and that relative to the identity line (*y* = *x*) is 0.65.

Lastly, the relationship between the sweat conductivity and the VC data was studied. The time interval of continuous flow of sweat through the reservoir was identified, and the continuous measurements at this time interval were compared for each patch location. Figure 4c shows the results at one location of participant 1. A significant relationship was found for all patch locations (mean *R*^2^ = 0.87), except for one case. In this case, the sweat rate did not increase significantly (Table S7). The results show that conductivity is directly related to sweat rate. When sweat rate increases, the relative ion reabsorption rate changes and sweat conductivity increases, which corresponds to the theory described by Baker and Wolfe.^20^ Thus, the patches can be used to accurately monitor sweat rate changes during exercise.

To summarize the results, the physiological experiments showed that the new patch can be used to test continuous sweat monitoring techniques and to simultaneously collect and store this sweat for separate, offline analysis. The sweat collection system enabled collection of 38 samples of 70 μL on average per participant during the physiological tests of 90 min. It was found that the [Na^+^] and [Cl^–^] of the samples were linearly related to conductivity, and conductivity data were related to VC sweat rate data as well. The current version of the patch was shown to work well, but the system can possibly be expanded with an extra sensor for semicontinuous absolute measurement of sweat rate with the patch. A new narrow microfluidic channel with a small collection surface could be integrated to minimize droplet formation and enclosure of air during sweat rate measurement. The filling of this new channel could be detected by placing tiny electrodes at multiple places along the channel and counting the moments that the fluid passes the channel, like in the patch of Yuan et al.^32^ The current filling rate electrodes should be maintained in this alternative version of the patch to use them for timing purposes.

In short, the new sweat patch facilitates real-time measurement and chronological sampling of sweat. The process is less labor-intensive per sample and less prone to contaminations during the protocol than alternative methods such as the absorbent patch technique. The simple fabrication process of the collection patch enables other researchers to easily replicate this system to compare their own sensors, such as electrochemical glucose sensors,^53^ or lactate sensors,^54^ against a reference. Figure S8 shows an example of how an external screen-printed potentiometric sensor can be integrated in the system. Furthermore, the reservoir volume and collection surface can be easily adapted to physiological tests performed in alternative conditions that evoke sweat rate levels of different orders of magnitude. In Figure S9, a technical drawing is shown which includes a system with reservoirs of 70 μL and a system with reservoirs of 130 μL. With this new patch, novel sweat sensors can be validated. Thereafter, the sensors can be used by physiologists to learn more about the mechanisms behind sweating and to identify new sweat biomarkers or markers for athlete performance monitoring.

## Conclusions

In this paper, we presented a new low-cost sweat collector patch for both continuous monitoring of analytes and chronological sampling of sweat for offline analysis. A conductivity sensor was integrated in the patch for continuous measurement, and level indicator electrodes measure the filling rate of the sweat in the sequence of reservoirs that store sweat for offline analysis. The new sweat patch was characterized in the lab. The sensitivity of the conductivity sensor was 24 μS/mM NaCl, and the reservoirs fill at the same speed as the sweat rate in the desired range (12 to 60 μL/min). In physiological experiments, continuous conductivity measurements were successfully compared to ventilated capsule (VC) sweat rate measurements and offline analysis of [Na^+^] and [Cl^–^] by ion chromatography. Results (*n* = 6, 3 locations for each participant) showed that VC sweat rate data were related to the conductivity measurements. Furthermore, sweat conductivity data were linearly related to [Na^+^] and [Cl^–^] from the samples stored in the reservoir sequence of the patch (*R*^2^ = 0.97). Thus, the new sweat collector patch allows to continuously measure sweat analyte concentrations during physiological experiments and to store the sweat in a sequence of reservoirs for offline reference measurement, as an *in situ* validation strategy for new sweat sensors.

## Supporting Information Available

Additional experimental results; data treatment; additional graphics, and technical drawings (PDF)
