# Supplementary material for: Low-Cost Wearable Fluidic Sweat Collection Patch for Continuous Analyte Monitoring and Offline Analysis
Source: Anal Chem. 2022 Apr 29;94(18):6893–901. doi: 10.1021/acs.analchem.2c01052 (PMC9096792; doi:10.1021/acs.analchem.2c01052)

Supplementary information for:

A LOW-COST WEARABLE FLUIDIC SWEAT COLLECTION PATCH FOR CONTINUOUS ANALYTE MONITORING AND OFFLINE ANALYSIS.

Annemarijn S.M. Steijlen*,†, Kaspar M.B. Jansen‡, Jeroen Bastemeijer†, Paddy J. French†, Andre Bossche†

†Delft University of Technology, Faculty of Electrical Engineering, Mathematics & Computer Science, Mekelweg 4, Delft, 2628 CD, The Netherlands. ‡Delft University of Technology, Faculty of Industrial Design Engineering, Landbergstraat 15, Delft, 2628 CE, The Netherlands.

**S1.**	**Performance of the ventilated capsule measurement system**	**2**
**S2.**	**Continuous heart rate & rectal temperature of participant 1**	**3**
**S3.**	**Participant during a physiological test**	**3**
**S4.**	**Raw sweat conductivity data over time for two participants**	**4**
**S5.**	**Relationship between [Na^+] and sweat conductivity and [Cl^-] and sweat conductivity**	**5**
**S6.**	**Data processing to find the patch filling rates**	**6**
**S7.**	**Squared correlation coefficients for ventilated capsule data and conductivity data**	**6**
**S8.**	**An example of how a novel sweat sensor can be integrated in the patch**	**7**
**S9.**	**Technical drawings of the sweat patches**	**8**

S1. Performance of the ventilated capsule measurement system

Method:

Lab measurements were performed to characterize the VC system. To find out if we can measure near real-time, it is important to study the response time and recovery time of the system. Absorption of moisture in the system may lower the response time. To quantify the response and recovery time, the cup was placed upside down and an accurately weighed amount of water (90 till 100 µl) was placed in the cup. The bottom of the cup was covered with a hydrophilic membrane (Merck-Millipore, USA) to ensure that the water maintains distributed over the entire surface. The capsule was sealed quickly and attached to the tubing. The capsule was attached to an aluminium U-profile, that was placed in water of 36 °C. Furthermore, it was verified if the total amount of water that was measured by the VC system corresponded to the amount of weighed water that was placed in the capsule. The total amount of sweat was calculated by numerical integration of the sweat rate curve.

Results:

A defined amount of water (~300 µl) was placed in the capsule, the capsule was covered and a measurement was performed till all water was evaporated. From the relative humidity readings, the evaporation rate was calculated and by numerical integration the total detected amount of water can be derived. The deviation from the initial amount of water was between 1% and 5%. A small deviation will always be present, because during weighing and during placement of the capsule in the setup, the water can evaporate for a short period of time (60-120 seconds). Figure S1.a) shows the response time and Figure S1.b) shows the recovery time of the ventilated capsule system at different flow rates. The numbers are shown in the table in Figure S1.c). At a flow rate of 0.6 l/min, the response time (which is defined as the time that it takes to reach 90% of the maximum pump rate) is 332 s and the recovery time is 685 s. At a flow rate of 0.6 l/min, a maximum evaporation rate of 8 mg/min can be reached. For the current capsule with a diameter of 2.6 cm, this means that the maximum sweat rate that can be measured is 1.7 mg/cm^2/min. When the flow rate is set at 1.2 l/min the response time and recovery time are 118 seconds and 353 seconds respectively and the maximum sweat rate that can be measured is 2.75 mg/cm^2/min. In the climate chamber, sweat rates at the back of the athlete can be larger than 2 mg/cm^2/min and it is important that all sweat is evaporated and measured. Therefore, the ventilated capsule measurement system will be set at a flow rate of 1.2 l/min. So, the response time will be less than 2 minutes. Since sweating is a reaction to increasing body temperature, sweat rates will always increase gradually, which means that the sensor will measure the right humidity value faster than the response time. In short, it can be concluded that the system can be used for continuous sweat rate monitoring.

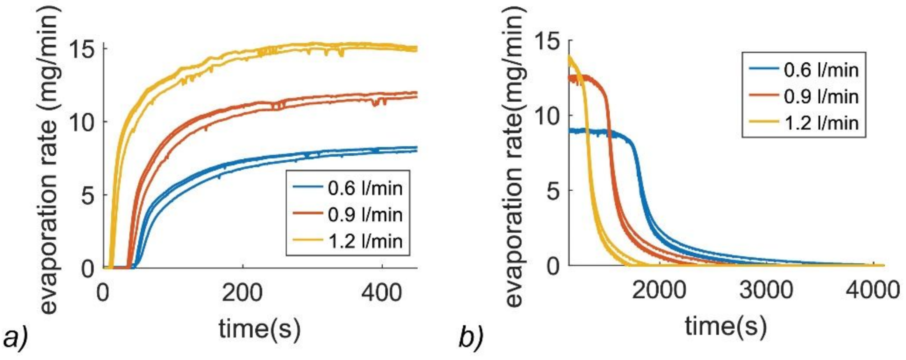

Air flowrate (l/min)	*Response time 63 % - 90 % (resp. in sec)*	*Recovery time 63 % - 90 % (resp. in sec)*
0.6 l/min	62-332	355-685
0.9 l/min	43-220	271-499
1.2 l/min	21-118	231-353

c)

Figure S1. Response time of the ventilated capsule: the evaporation rate calculated from the relative humidity measurements plotted over time at different flow rates of the dry air (measurements with 3 humidity sensors are plotted) in a) the response time is shown and in b) recovery time is shown. c) Table with response time and recovery time data.

Figure S2. Continuous heart rate measurement of participant 1 (top) and continuous measurement of rectal temperature of participant 1 (bottom).

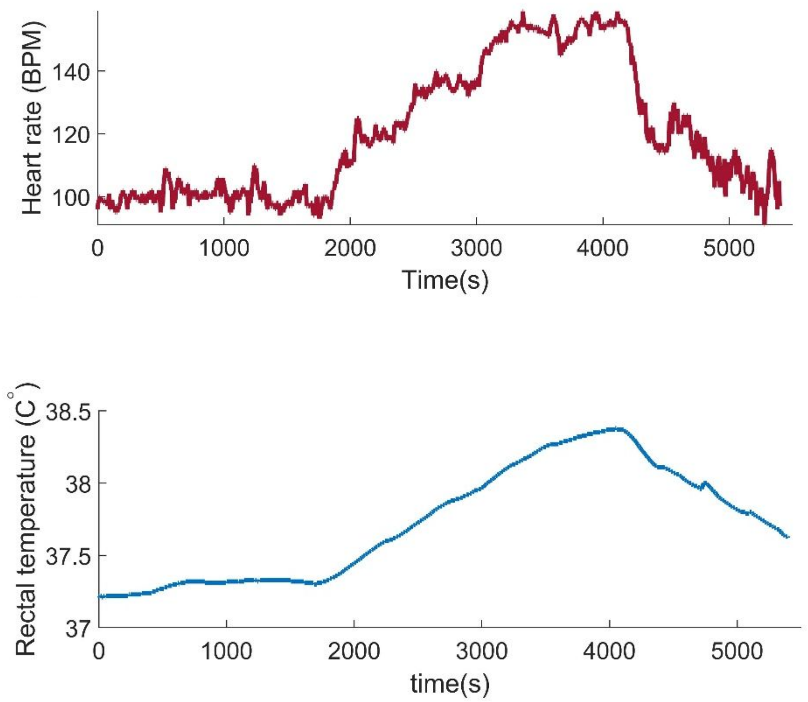

Figure S3. Participant during a physiological test. Patches are placed at the left and the blue capsules are placed at the right.

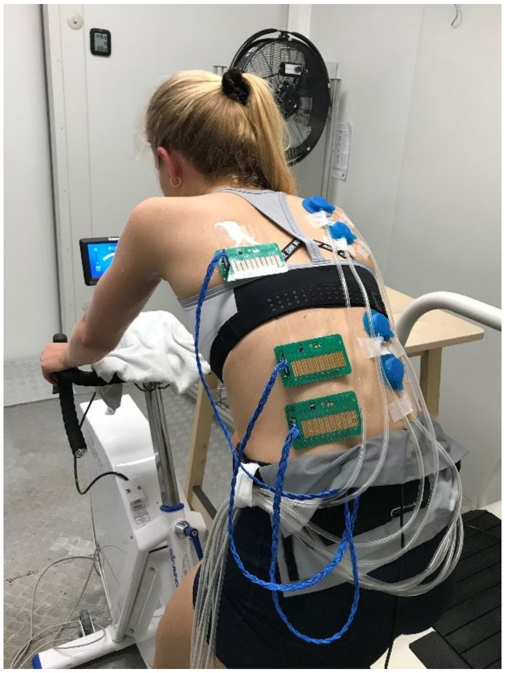

Figure S4. Raw sweat conductivity data over time for two participants. The blue, yellow and orange lines show the raw data of the conductance measurements at the 3 locations. The black lines are the filtered data. First, conductances below $0.5*10^3$ S were removed, because this indicates that there is air in the chamber. Thereafter, the data were filtered using a Savitsky-Golay sliding window filter (window size = 50 samples and a second order polynomial was chosen. The black dots are the data points that are used in the comparison with the measurements from the ion chromatograph.

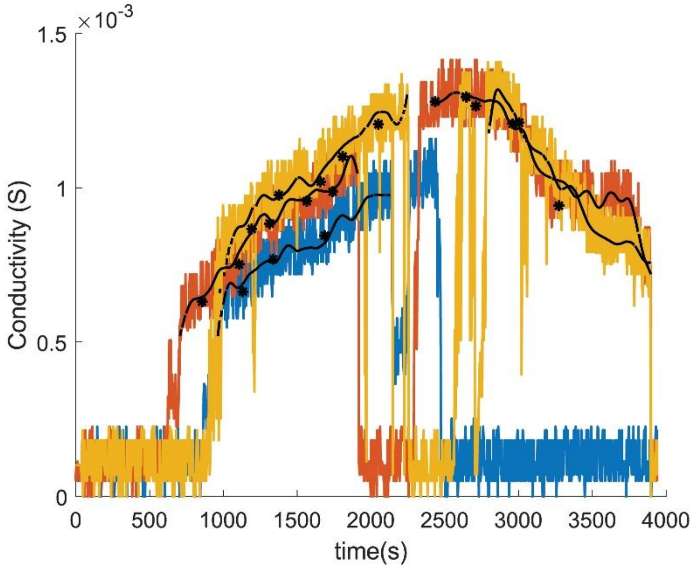

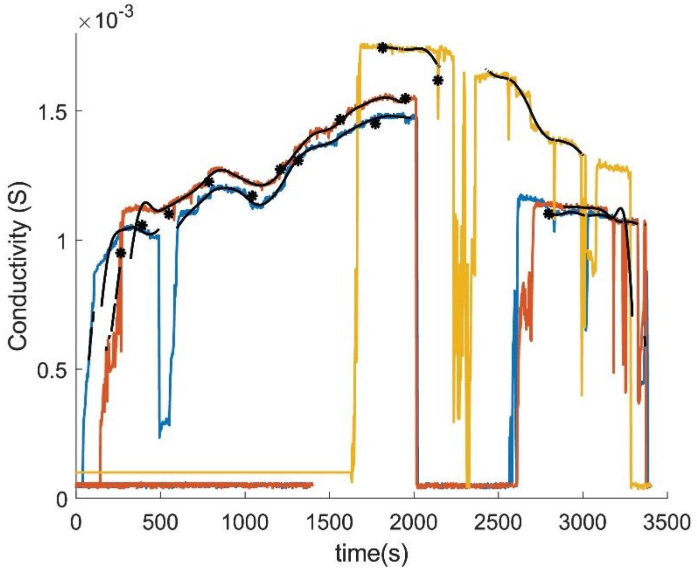

Figure S5. Relationship between [Na^+] and sweat conductivity (left) and [Cl^-] and sweat conductivity (right) at all three locations for each participant. For participant 7, less than two samples per location were collected, because of exceptionally low sweat rates. These results were not shown here. Error bars show the mean root mean square errors between the filtered conductance data and the raw data.

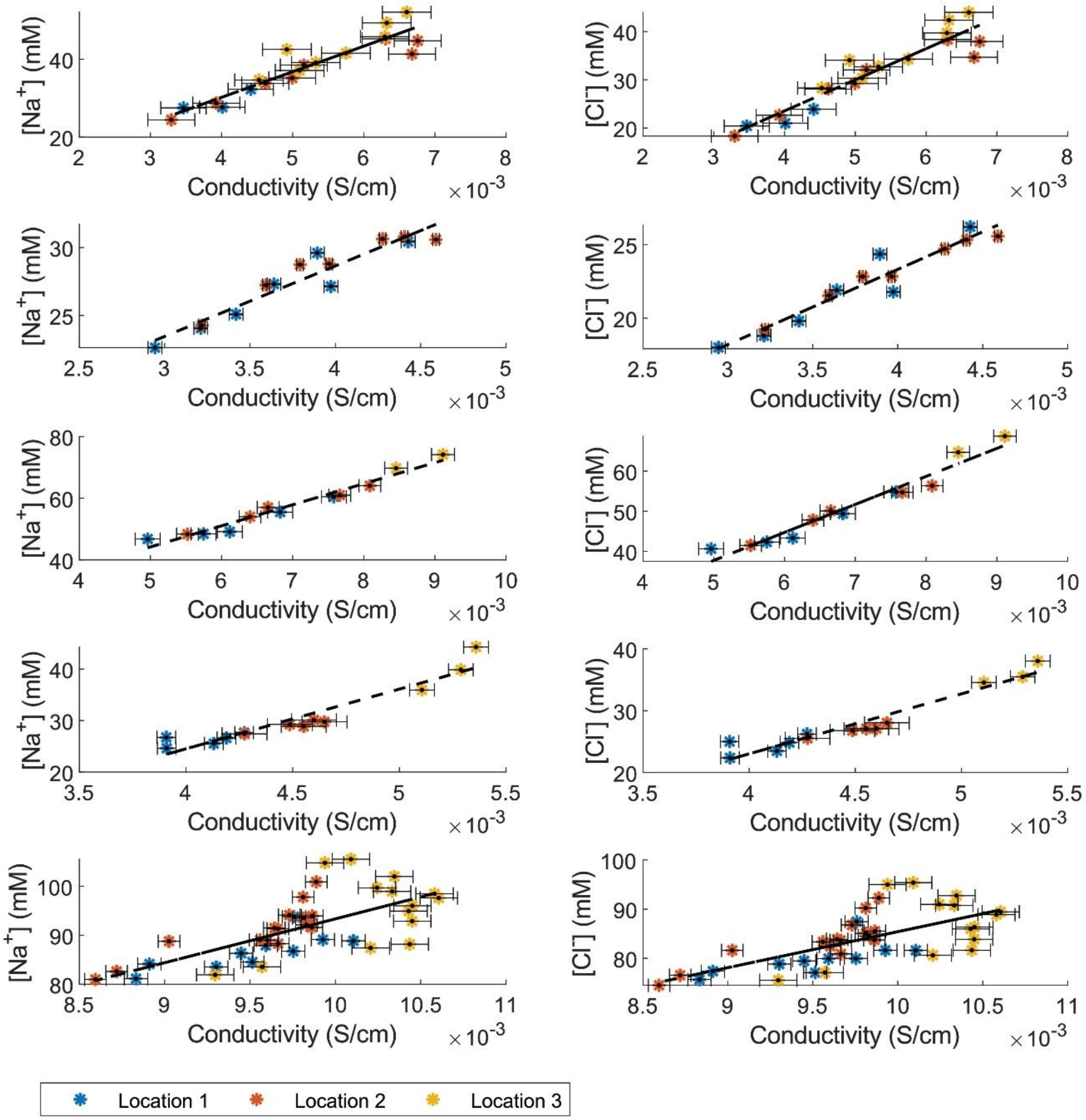

Figure S6. Data processing to find the patch filling rates: an example with two collectors of participant 4 a) the conductance change for each electrode pair. b) Cumulative conductance change after removal of the conductances below $0.5*10^{-3}$ S c) Calculated filling rate including the linear fit that was used to derive the average filling rate. The abrupt changes in conductance originate from droplets that are formed and drop down into the reservoir. Sweat rates that are calculated for location 1 (blue) and location 2 (orange) are 1.19 and 1.62 mg/cm^2/min.

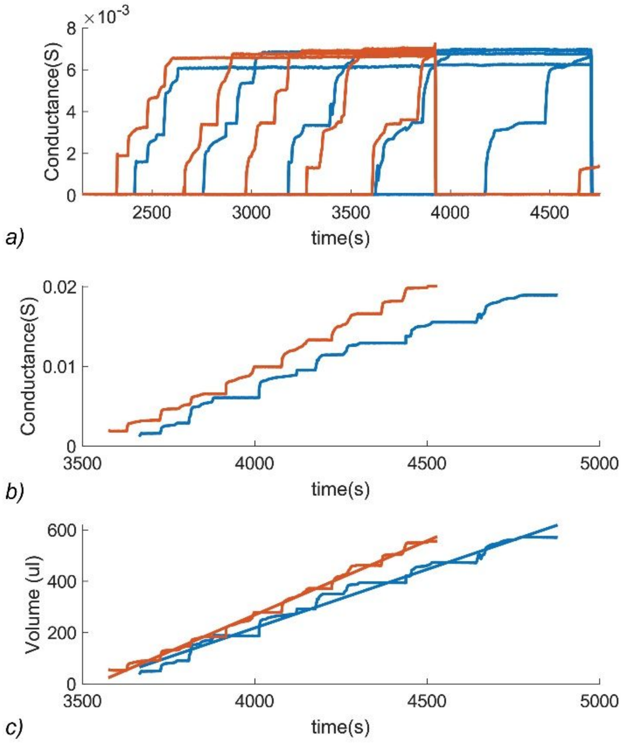

Table S7. Squared correlation coefficients for ventilated capsule data and conductivity data for the different participants and patch locations. *At patch location 3 of participant 4, sweat rates did not increase significantly leading to a low R^2 value

Participant	*Collector location*	*R^2*
P1	1	0.94
	2	0.90
	3	0.95
P2	2	0.71
P3	1	0.88
	2	0.82
	3	0.83
P4	1	0.87
	2	0.92
	3	0.27*

Figure S8. An example of how a novel sweat sensor can be integrated in the patch for validation experiments in a physiological setting: A screen-printed potentiometric sensor is placed between the PCB layer and the spacer tape. The PCB layout is adapted, so that a specific connector, such as an FFC/FPC connector, can be used. Signals can be read out via similar header pins as used for the conductivity readout.

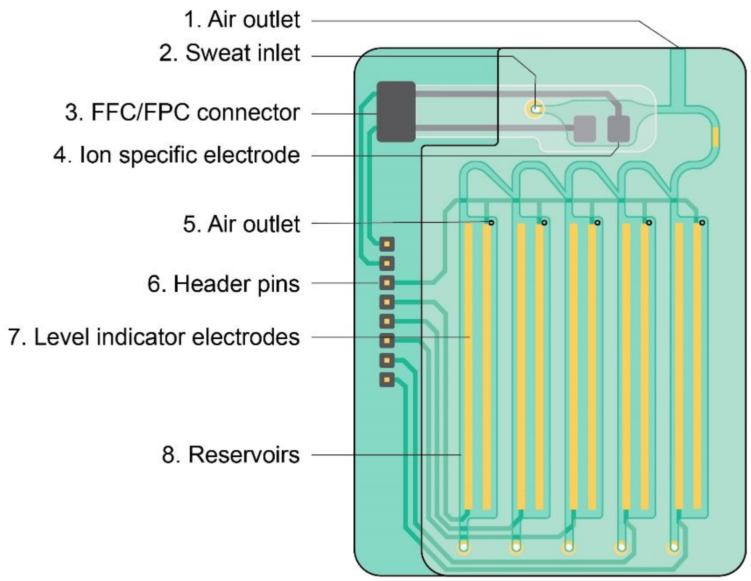

Figure S9. Technical drawings of the sweat patches used in the experiments. The patch with 10 reservoirs (left) and the patch with 5 reservoirs (right). The spacer tape consists of 4 layers of adhesive tape that are laminated to obtain the desired reservoir width (0.64 mm).

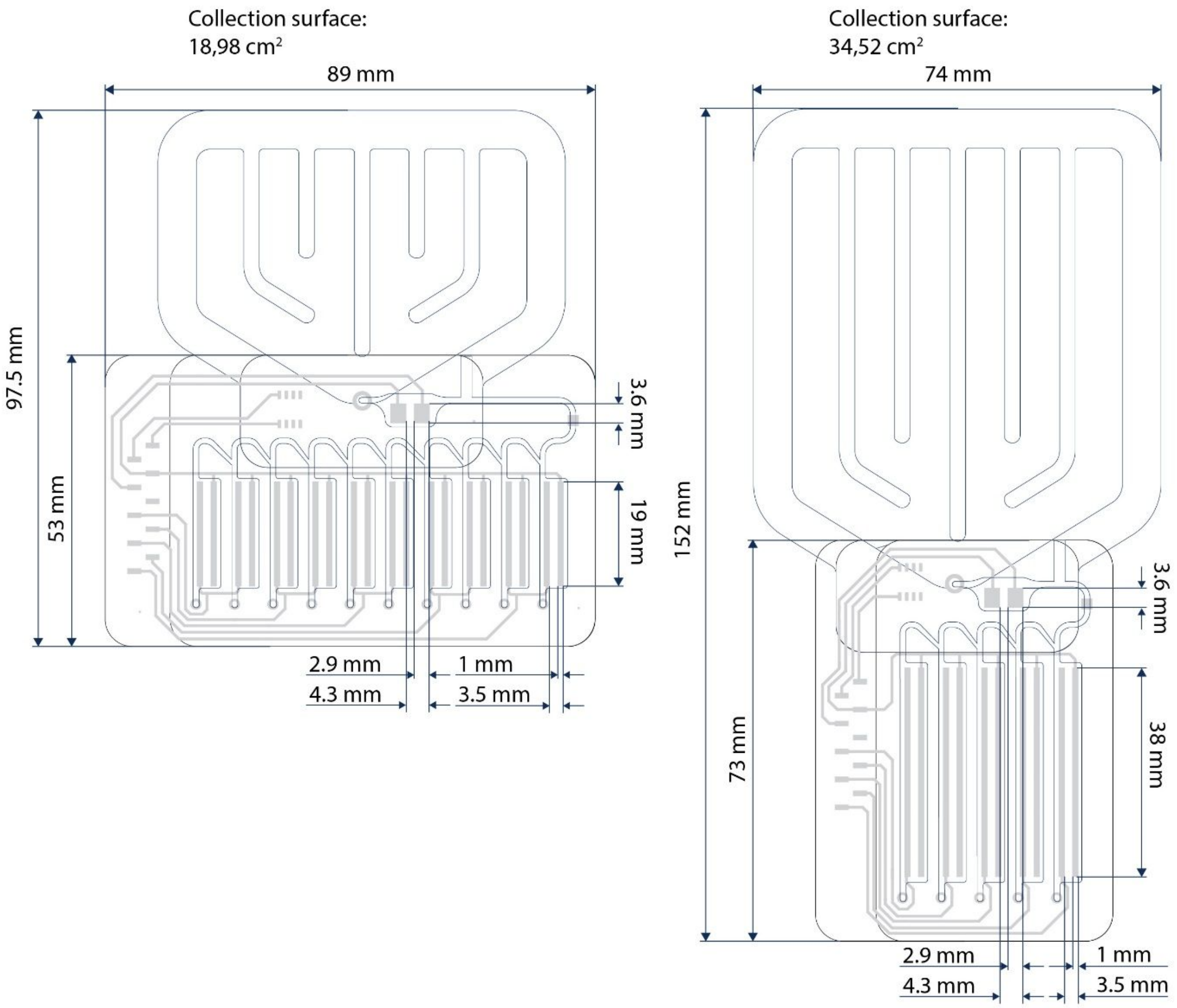

Supplement: Supplementary file 1 — ac2c01052_si_001.pdf [file ac2c01052_si_001.pdf]
